# Temporal Shotgun Metagenomics Revealed the Potential Metabolic Capabilities of Specific Microorganisms During Lambic Beer Production

**DOI:** 10.3389/fmicb.2020.01692

**Published:** 2020-07-17

**Authors:** Jonas De Roos, Marko Verce, Stefan Weckx, Luc De Vuyst

**Affiliations:** Research Group of Industrial Microbiology and Food Biotechnology (IMDO), Department of Bioengineering Sciences, Vrije Universiteit Brussel, Brussels, Belgium

**Keywords:** shotgun metagenomics, *Dekkera*/*Brettanomyces*, malolactic fermentation, maltooligosaccharides, esterases, mass spectrometry

## Abstract

Lambic beer production processes are characterized by a temporal succession of well-adapted microbial species. Temporal metagenomic analysis of a Belgian, traditional, lambic beer production process, which was examined microbiologically and metabolomically before, confirmed that the microbial diversity is limited. Moreover, it allowed to link the consumption and production of certain compounds to specific microbial groups or species. Fermentation characteristics, such as the conversion of malic acid into lactic acid and acetoin production, were retrieved and could be attributed to specific microorganisms, namely *Pediococcus damnosus* and *Acetobacter* species, respectively. Traits previously ascribed to brewery-specific *Dekkera bruxellensis* strains were confirmed during the lambic beer production process examined multiphasically; in particular, the higher production of 4-ethylguaiacol compared to 4-ethylphenol was further shown by mass spectrometric analysis. Moreover, the absence of phenolic acid decarboxylase in *Brettanomyces custersianus* was shown culture-independently and could explain its late occurrence during the maturation phase. Furthermore, the potential of maltooligosaccharide degradation could be ascribed metagenomically to not only *Brettanomyces* species but also *Saccharomyces kudriavzevii*, possibly explaining their degradation early in the lambic beer production process. Also, acetic acid bacteria (AAB) seemed to be able to consume maltooligosaccharides via their conversion into trehalose. Furthermore, these AAB possessed esterase genes, potentially capable of forming ethyl acetate, which may contribute to the flavor of lambic beer. Improved knowledge on the reasons behind certain community dynamics and the role of the different microorganisms in terms of potential functionality could improve brewery practices to assure to produce more quality-stable end-products.

## Introduction

Belgian lambic beers rely on a long production process in horizontal wooden barrels, which renders them refreshingly acidic with very little residual carbohydrates (Spitaels et al., 2017; De Roos and De Vuyst, 2018; De Roos et al., 2018a, b, 2019). Due to their acid-refreshing character and fruity notes, acidic ales are witnessing an increasing popularity worldwide (Pothakos et al., 2016; Spitaels et al., 2017). Although they are mostly produced in traditional breweries, Belgian-style acidic ales manufactured in common breweries, such as American coolship ales (ACAs) produced in the United States, appear as well (Bokulich et al., 2012; Spitaels et al., 2014b, 2015). The fermentation and maturation process of Belgian lambic beers is initiated by environmental inoculation and proceeds for up to 3 years, thereby going through four distinct phases comprised of different microbial groups, namely the enterobacterial phase [enterobacteria, acetic acid bacteria (AAB), and oxidative yeasts], the alcoholic fermentation phase (species of the fermentative yeast genus *Saccharomyces*), the acidification phase [in particular, the lactic acid bacteria (LAB) species *Pediococcus damnosus* and, to a lesser extent, the AAB species *Acetobacter lambici* and *Acetobacter pasteurianus*], and the maturation phase (in particular yeast species belonging to the *Brettanomyces* genus) (Spitaels et al., 2014b, 2015, 2017; De Roos and De Vuyst, 2018; De Roos et al., 2018a, b). As lambic beers represent this diverse microbial ecosystem, most former studies focused on culture-dependent microbiological analyses, whether or not in combination with restricted metabolite target analyses (Van Oevelen et al., 1976, 1977; Spaepen et al., 1978, 1979; Verachtert and Dawoud, 1984; Verachtert et al., 1989; Martens et al., 1991, 1992; Shanta Kumara and Verachtert, 1991; Spitaels et al., 2014b, 2015).

Studies relying solely on microbiological plating have some drawbacks and can be strengthened by a culture-independent analysis. For example, the selection of isolation media to cultivate specific microorganisms is based on *a priori* knowledge about the microbial communities present in the ecosystem, which can lead to biased results (Nocker et al., 2007). Besides possibly leading to a distorted view of the microbial communities present, some of the microorganisms can also be in a viable but not culturable state, making their detection with culture-dependent techniques nearly impossible. Moreover, the microbial species that comprise the culturable part of the ecosystem under study can have varying generation times, which endangers the isolation of slow-growing species that are outcompeted easily (Nocker et al., 2007). Therefore, amplicon sequencing of whole-community DNA has been applied to get insight into the microbial species diversity of ACAs (Bokulich et al., 2012) and Belgian lambic beers (De Roos et al., 2018a, 2019). Amplicon sequencing is widespread to study fermented food ecosystems (De Filippis et al., 2017).

Although the microbial successions during lambic beer production processes have been described through a combination of physiological, high-throughput microbiological, and metabolomic analyses, the role of these microorganisms often remains undetermined in terms of functionality and interactions (De Roos et al., 2018a, b). As shotgun metagenomics relies on high-throughput sequencing of whole-community DNA, it enables the *in silico* study of the diversity and metabolic potential of the different microbial groups present in an ecosystem. Indeed, being a culture-independent technique, it circumvents the problem of *a priori* knowledge of the microbial members involved, as is the case for culture-dependent and PCR-based techniques (Illeghems et al., 2012; Ercolini, 2013). Moreover, metagenomic data enable the retrieval of the functional potential of all microorganisms present in an ecosystem, without the need for prior knowledge of the exact sequences of the genes involved, which is, however, the case with PCR screenings (Petrosino et al., 2009; Illeghems et al., 2012; Ercolini, 2013). Additionally, as shotgun metagenomic sequencing is performed on whole-community DNA, directly extracted and purified from environmental samples, without a prior PCR amplification step, it omits PCR-based errors (Sipos et al., 2007; Gonzalez et al., 2012; Větrovský and Baldrian, 2013; Sternes et al., 2017).

In contrast to amplicon sequencing, shotgun metagenomic studies of fermented food ecosystems with a concomitant functional analysis based on gene prediction are much less applied (Escobar-Zepeda et al., 2016; Wu et al., 2017; Pothakos et al., 2020). However, it seems to be a robust tool for assessing both the species diversity and functionality of microorganisms in various fermented foods and beverages. Regarding beers, shotgun metagenomics has only been applied on one sample of a spontaneously inoculated ACA production batch, thereby focusing primarily on the taxonomy of the microbial ecosystem (Heil et al., 2018). Therefore, the present study aimed to use this approach to assess both the taxonomy and functionality of the microorganisms present in a traditional Belgian lambic beer production process. This particular production process has been well-characterized before in terms of microbial species diversity, microbial community dynamics, and substrate consumption and metabolite production kinetics through targeted microbiological and metabolomic approaches (De Roos et al., 2018a, b). A combination of all these data will enable to assess not only the accuracy of the metagenomic approach but also the possibility to ascribe potential functional properties unraveled through metagenomics to actual substrate consumption and metabolite production kinetics determined phenotypically during the successive microbial phases of the lambic beer fermentation and maturation process.

## Materials and Methods

### Lambic Beer Production and Sampling

Wort of 12.6°P, prepared according to the brewer’s recipe in a Belgian traditional lambic brewery, was used for a long-term fermentation and maturation process in wooden casks. This lambic beer production process was previously sampled and assessed with various microbiological and metabolomic analysis methods (De Roos et al., 2018a, b). During the present study, the same samples coming from the same lambic beer production process were subjected to a temporal metagenomic analysis. Therefore, the cooled samples, taken aseptically from the fermenting wort and maturing lambic beer after 3 days (further referred to as B3D); 3 weeks (B3W); and 3 (B3M), 9 (B9M), 13 (B13M), and 24 (B24M) months after the transfer of the cooled wort to the wooden casks, were subjected to a centrifugation step (4,618 × *g*, 20 min, 4°C) to obtain cell pellets and cell-free supernatants as described before (De Roos et al., 2018a, b). Pellets and supernatants were stored at −20°C and used for metagenomic analysis and dedicated metabolite target analysis (metabolites not measured before), respectively. Microbiological analysis (including DNA isolation and high-throughput sequencing) and metabolite target analysis were performed after sampling as soon as possible. The bioinformatic analysis of the different metagenomic data sets was performed all at once after the lambic beer production process ended to avoid biases due to changing databases.

### Metagenomic DNA Extraction

To enable a culture-independent metagenomic analysis, total DNA extracts were prepared from the cell pellets obtained from the samples B3D, B3W, B3M, B9M, B13M, and B24M to get six metagenomes. The metagenomic DNA extraction was carried out in tubes and comprised enzymatic, chemical, and mechanical treatments of the cell pellets mentioned above to lyse the cells, followed by phenol/chloroform/isoamyl alcohol treatment and column purification to extract and purify the DNA, as described previously (Vermote et al., 2018). However, the elution from the DNeasy Mini Spin columns (Qiagen, Hilden, Germany) was performed with two times 100 μl of nuclease-free water (Amresco, Solon, OH, United States) to recover as much metagenomic DNA as possible. Then, the eluates were treated with 4 μl of RNase (10 mg/ml; Roche, Basel, Switzerland) at 37°C for 10 min, again followed by purification with the DNeasy Blood & Tissue Kit (Qiagen) according to the manufacturer’s instructions. After performing the final elutions with 100 μl of nuclease-free water (Amresco), the overall quality and the absence of RNA after RNase treatment of the extracted DNA were assessed by agarose gel electrophoresis. Finally, a NanoDrop 2000 spectrophotometer and a Qubit 2.0 fluorometer using a Qubit dsDNA HS Assay Kit (all from Thermo Fisher Scientific, Wilmington, Germany) were used to measure the DNA concentrations.

### Preparation of DNA Libraries, Shotgun Metagenomic Sequencing, and Data Pre-processing

DNA library preparation and sequencing was based on a methodology described before (Vermote et al., 2018; Verce et al., 2019). All materials and apparatus were from Thermo Fisher Scientific and all protocols were performed according to the manufacturer’s instructions, unless stated otherwise. The metagenomic DNA was enzymatically sheared using an Ion Xpress Plus Fragment Library Preparation Kit to produce DNA library fragments of the desired length (approximately 400 base pairs or bp). The shearing time was first optimized with 100 ng of metagenomic DNA as input. Afterward, a shearing reaction was performed using the same shearing protocol (Ion Xpress Plus Fragment Library Kit). The resulting sheared DNA underwent adapter ligation and nick repair as well as multiple purifications. This resulted in six 400-bp size-selected libraries that were qualitatively and quantitatively checked before being used as a template for emulsion PCR onto Ion Sphere Particles (ISPs). The enriched template-positive ISPs were loaded on an Ion 316 Chip v2 for DNA sequencing using an Ion Hi-Q Sequencing Kit on an Ion PGM. The metagenomic sequence data were quality checked and trimmed as described before (Verce et al., 2019).

### Taxonomic Analysis of the Metagenomic Sequence Data

The taxonomic analysis of the metagenomic sequence data of the lambic beer production samples was performed by using various software packages and databases to avoid software and database biases, so that the results did not depend on a single taxonomic profiling tool (Vermote et al., 2018; Verce et al., 2019).

#### Taxonomic Analysis Using All Metagenomic Sequence Reads

The taxonomic composition of the lambic beer production samples was assessed, using the quality-trimmed metagenomic sequence reads, which were analyzed with various taxonomy profiling tools, namely the basic local alignment search tool (BLAST; Altschul et al., 1990), Kraken (Wood and Salzberg, 2014), and Kaiju (Menzel et al., 2016). Sequences from a non-redundant nucleotide (nt) database of the National Center for Biotechnology Information (NCBI, Bethesda, MA, United States)^1^, together with a custom-made database consisting of bacterial, archaeal, and fungal genomic sequences obtained from RefSeq (NCBI; Tatusova et al., 2014), were used to align the metagenomic sequence reads with the BLAST algorithm nucleotide blast (blastn). Sequences from the NCBI non-redundant protein (nr) database were used to align the metagenomic sequence reads with Diamond (Buchfink et al., 2015). Both outputs of Diamond and blastn were parsed with Megan 5.7.0 (Huson et al., 2011), using the following settings: minimum score, 100; maximum expected value, 0.01; percentage of top hits taken into account for the analysis, 10.0; minimum support value, 150; and lowest common ancestor (LCA) percentage, 100. The results were further processed and visualized with R (R Core Team, 2017) in RStudio (RStudio Team, 2015), using the packages ggplot2 (Wickham, 2009) and tidyverse (Wickham, 2017).

Also, sequences of complete bacterial, archaeal, and fungal genomes from NCBI GenBank were used to construct a custom-made database for sequence classification using Kraken version 0.10.5-beta. A custom-made database consisting of prokaryotic and microbial eukaryotic protein sequences from the nr database was used for sequence classification with Kaiju.

#### Taxonomic Analysis Based on Metagenomic Recruitment Plotting

For species-level taxonomic analysis of the metagenomes of the lambic beer production samples, metagenomic recruitment plots were constructed using genera that were represented by more than 0.1% of all metagenomic sequence reads, as found with any alignment-based method, in any of the six metagenomes (Vermote et al., 2018; Verce et al., 2019). Temporal shifts in the microbial communities of the lambic beer production process could be followed using these metagenomic recruitment plotting results. In short, the NCBI RefSeq assembly database was used to obtain genome sequences of the sequenced type strains from the genera that were selected. The metagenomic sequence reads were used as query sequences, to which a BLAST search was performed using blastn and the collected genome sequences as database. The minimum identity threshold was set to 60% and only the top hit for each sequence was retained, using 60% as the minimum query coverage threshold.

### Metagenomic Sequence Read Assembly, Assignment of Contigs to Taxa, and Contig Clustering

Functional analysis of the metagenomes of the six lambic beer production samples was based on contig assembly and gene prediction, as described previously (Verce et al., 2019). The MEGAHIT assembler (Li et al., 2015), with the preset parameter set “meta-sensitive,” was used on a data set consisting of all sequence reads of the six metagenomes to assemble the metagenomic sequence reads into contigs. Only contigs of at least 1,000 bp were retained. By using an in-house developed Python script, various assembly statistics, including total size of the assembly (*in casu* 73.10 Mbp), the number of contigs (16,493), the longest contig length (155,982 bp), the average contig length (4,432 bp), the median contig length (2,664 bp), and the N50 value (6,642 bp) were calculated. The contigs were mapped to reference genomes with BWA MEM (Li, 2013) and binned with CONCOCT into 29 bins (Alneberg et al., 2014). The quality of the metagenomic bins, including completeness, contamination, and strain heterogeneity were checked using CheckM v1.0.9 (Parks et al., 2014). These bins could be regarded as comprising contigs from either one or several microbial species. In contrast, contigs of one species could be assigned to different bins, which was, for example, the case for the yeast species. All bins could be associated with at least one of the bacterial/yeast species, leaving no cluster unaccounted for. The combined sizes of contigs in bins attributed to certain species were similar to the sizes of their respective representative genomes, as was the case for *Dekkera bruxellensis* (12.9 vs. 13.4 Mbp), *Saccharomyces cerevisiae* (10.5 vs. 12.2 Mbp), *Saccharomyces kudriavzevii* (10.5 vs. 8.7 Mbp of a scaffold genome), *Pichia membranifaciens* (10.7 vs. 11.6 Mbp of a scaffold genome), and *P. damnosus* (2.4 vs. 2.5 Mbp). The *Acetobacter* bin contained contigs from at least two species, as shown by its size (7.1 vs. 3.0 Mbp of the *A. pasteurianus* genome). Its contigs mapped to *A. pasteurianus* and several different *Acetobacter* species, as analyzed by BWA MEM. According to CheckM, the *Acetobacter* bin showed 98.28% completeness and 95.92% contamination. In contrast, the *P. damnosus* bin showed 95.06% completeness and 0% contamination, and the *Klebsiella* bin showed only 6.28% completeness and 0% contamination. For assessment of the fungal bins, CheckM was not appropriate, as this tool relied on bacterial marker genes solely.

### Functional Analysis of the Lambic Beer Metagenomes

The actual functional analysis of the metagenomes of the six lambic beer production samples was performed as described previously (Verce et al., 2019). A custom-build database using bacterial, archaeal, and fungal protein sequences from Swiss-Prot was used with Prokka to annotate the assembled metagenomic contigs (Seemann, 2014). The resulting Prokka annotations served as the basis for a targeted search of relevant metabolic pathways. The predicted products of coding sequences found in the contigs were screened with HMMER 3.1b1^2^ using the dbCAN database (Yin et al., 2012). CAZy (Lombard et al., 2014) and Cazypedia, the encyclopedia of carbohydrate-active enzymes^3^, were used as references. This facilitated the search for carbohydrate-active enzymes. The features that were found during the examination of the contigs were linked to the species found by the taxonomic analysis through mapping of the contigs to the genomes of said species with BWA-MEM. In addition, the binning with CONCOCT was used to facilitate the identification of those contigs that were not mapped to any of the relevant genomes. The presence of genes encoding some specific enzymes was assessed by performing alignment searches using protein blast (blastp; NCBI), whereby their amino acid sequences were used as query sequences and the amino acid sequences derived from the metagenomic assembly as database. This was the case for genes encoding biogenic amine-forming decarboxylases, two specific β-glucosidases, phenolic acid decarboxylases, vinyl phenol reductases, hop resistance genes, *Acetobacter*-specific esterases, and the gene supposedly responsible for the ropy phenotype in *P. damnosus*.

### Accession Number

All six metagenomic data sets were submitted to the European Nucleotide Archive of the European Bioinformatics Institute (ENA/EBI) and are accessible under the study accession number PRJEB28363^4^.

### Metabolite Target Analysis

Cell-free supernatants were used to determine the concentrations of dedicated metabolites. All samples were both prepared and analyzed in triplicate.

#### Determination of Lactic Acid Stereoisomer Concentrations

The concentrations of D-lactic acid and L-lactic acid were measured by high-performance liquid chromatography with ultraviolet detection (HPLC-UV) and external calibration, as described previously (De Roos et al., 2018a).

#### Determination of 2-Methylbutanol and 3-Methylbutanol Concentrations

The concentrations of 2-methylbutanol and 3-methylbutanol were measured by static headspace analysis via gas chromatography with mass spectrometric detection (SH-GC-MS). Therefore, headspace vials containing 2 ml of sample were analyzed with an Agilent 6890 gas chromatograph coupled to an Agilent 5973N mass spectrometer (Agilent Technologies), equipped with an MPS2 Gerstel autosampler (Interscience, Breda, The Netherlands) and an apolar DB-5ms column (15 m × 0.25 mm × 0.25 μm; Agilent Technologies). Samples were equilibrated by agitation at 45°C for 45 min prior to split injection (split ratio of 10) with a split-splitless injector at 250°C. The injection volume was set at 1 ml and the oven temperature program consisted of an initial step at 40°C for 1.5 min, followed by a linear increase from 40 to 230°C at 10°C/min. Finally, the temperature remained constant at 230°C for 4 min. The temperature of the transfer tube was held at 280°C. A constant carrier flow (1 ml/min) of helium gas was maintained during GC analysis. Ions were obtained via electron impact ionization at 70 eV and a temperature of 280°C. Full scan analysis with a scan range of a *m/z* value of 45–250 was used. Quantification was performed using external calibration.

#### Determination of 4-Ethylguaiacol and 4-Ethylphenol Concentrations

The concentrations of the phenolic compounds 4-ethylguaiacol and 4-ethylphenol were measured by static headspace analysis via gas chromatography coupled to tandem mass spectrometry (SH-GC-MS/MS). A Trace 1300 gas chromatograph coupled to a TSQ 8000 Evo triple quadrupole mass spectrometer, equipped with a Triplus RSH autosampler (Thermo Fisher Scientific, Austin, TX, United States) and a polar Stabilwax-MS column (30 m × 0.25 mm × 0.25 μm; Restek, Bellefonte, PA, United States) was used. Samples were equilibrated by agitation at 45°C for 45 min prior to split injection (split ratio of 15) with a split-splitless injector at 250°C. The injection volume was set at 1.2 ml and the oven temperature program consisted of an initial step at 40°C for 1.5 min, followed by a linear increase from 40 to 230°C at 10°C/min. Finally, the temperature remained constant at 230°C for 4 min. The temperature of the transfer tube was held at 280°C. A constant carrier flow (1 ml/min) of helium gas was maintained during GC analysis. Ions were obtained via electron impact ionization at 70 eV and a temperature of 280°C. Full scan analysis with a scan range of a *m/z* value of 45–250 was used, simultaneously with selected reaction monitoring (SRM). One quantifier and two qualifier mother and daughter ion combinations were followed for 4-ethylguaiacol and 4-ethylphenol. These combinations were 137 > 94, 137 > 122, and 152 > 137 for 4-ethylguaiacol, and 107 > 77, 122 > 77, and 122 > 107 for 4-ethylphenol. Quantification was performed using these SRMs and a standard addition method. Therefore, 50 μl of each standard solution (std A, std B, std C, std D, or std E) was added to a separate headspace vial containing 1,950 μl of sample. The five standard solutions contained the following compositions: solution A consisted of ethanol (5.0%, v/v) and solutions B, C, D, and E contained 12.5, 25.0, 50.0, and 100.0% of a stock solution of both 4-ethylguaiacol and 4-ethylphenol in 5% (v/v) ethanol. Calibration curves and effective concentrations of the compounds targeted within the fermenting matrix were calculated using the Tracefinder software (Thermo Fisher Scientific).

### Statistics

A Spearman correlation analysis (*P* < 0.05) between the different microbial species present and the concentrations of metabolites produced was performed, as described before (Bertuzzi et al., 2018). For the microbial species, the relative abundances found by taxonomic analysis based on metagenomic recruitment plotting were taken into account. Intra-sample diversity (alpha-diversity) was assessed by calculating the Simpson (diversity) and Pielou (evenness) indexes based on the genus-level results obtained from the metagenomic recruitment plotting analysis.

## Results

### Metagenomic Sequencing

The sequencing of the six metagenomic libraries derived from samples of a lambic beer production process taken as a function of time, corresponding with 3 days (B3D), 3 weeks (B3W), and 3, 9, 13, and 24 months (B3M, B9M, B13M, and B24M, respectively), resulted in six data sets with a combined size of 3.67 Gbp after quality trimming.

#### Taxonomic Analysis of the Lambic Beer Metagenomes

##### Taxonomic analysis using all metagenomic sequence reads

**FIGURE 1:**
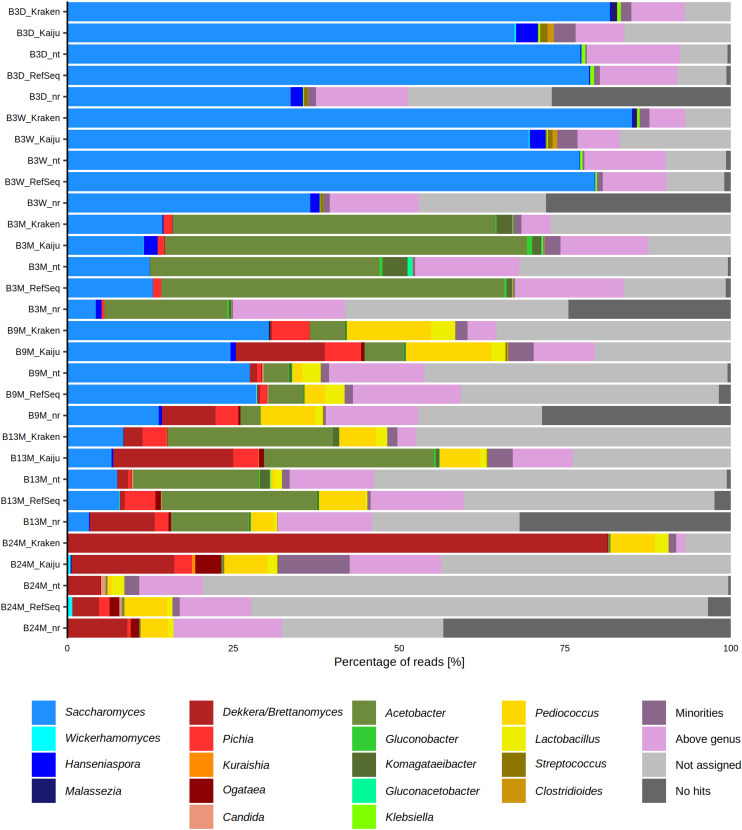
Percentage of metagenomic sequence reads from six metagenomes of a 24-month lambic beer production process carried out in wooden casks assigned to different genera, using different taxonomy profiling tools, namely BLAST (NCBI nucleotide database nt and RefSeq), Diamond (NCBI protein database nr), Kraken, and Kaiju. The category “Minorities” includes all genera that were represented by less than 0.5% of all metagenomic sequence reads. The category “Above genus” represents all assigned taxonomic levels higher than the genus level. The category “Not assigned” represents BLASTn and DIAMOND hits that did not fit the criteria from MEGAN. This category was also produced by Kraken and Kaiju for reads that produced no hits with these two profiling tools. The category “No hits” includes the reads that did not produce any hits with BLASTn and Diamond. This category was specific for these two profiling tools. The sample codes represent fermentation and maturation samples withdrawn after 3 days (B3D), 3 weeks (B3W), 3 months (B3M), 9 months (B9M), 13 months (B13M), and 24 months (B24M).

The six metagenomic sequence data sets were subjected to different taxonomy profiling tools to assess the microbial composition of the lambic beer fermentation and maturation samples. This resulted in slightly differing taxa and varying percentages of metagenomic sequence reads, according to the different taxonomy profiling tools used, showing the importance of using multiple tools for taxonomic analysis (Figure 1).

The most abundant yeast genera in the six metagenomes were, in decreasing order of relative abundances, *Saccharomyces*, *Brettanomyces*, *Pichia*, *Hanseniaspora*, *Ogataea*, *Candida*, *Kuraishia*, *Malassezia*, and *Wickerhamomyces*. Based on these metagenomic data, species belonging to the *Saccharomyces* genus were abundantly present during the first weeks of fermentation (between 60 and 90% of the metagenomic sequence reads). Species belonging to the *Hanseniaspora* genus could only be retrieved from the B3D and B3W samples and were present in much lower relative abundances than the *Saccharomyces* species (less than 4% of the reads). Toward the end of the lambic beer production process, the prevalence of species belonging to the genera of *Brettanomyces*, *Ogataea*, *Pichia*, *Candida*, and *Wickerhamomyces* increased, with *Brettanomyces* as the most prevalent yeast genus after 24 months of maturation (up to 90% of the reads).

The most abundant bacterial genera in the six metagenomes were, in decreasing order of relative abundances, *Acetobacter*, *Pediococcus*, *Komagataeibacter*, *Lactobacillus*, and *Klebsiella*. At the beginning of the fermentation, species belonging to the *Klebsiella* genus represented the most prevalent bacteria (less than 0.7% of the reads). At month 3 of the lambic beer production process, almost all metagenomic sequence reads could be ascribed to genera belonging to the AAB, with species representing the genus *Acetobacter* being most prevalent (more than 40% of the reads). During the maturation phase, the relative abundances of species belonging to the LAB genera *Lactobacillus* and *Pediococcus* increased (up to 25% of the reads), whereby species belonging to the *Acetobacter* genus remained present.

Taking into account both bacterial and yeast genera throughout the lambic beer production process, it turned out that yeast species belonging to the genus *Saccharomyces* were most prevalent at the beginning of the fermentation (B3D and B3W samples). After this yeast fermentation phase, species belonging to the *Acetobacter* genus became the most prevalent (B3M sample). During the maturation phase (B9M, B13M, and B24M samples), *Brettanomyces*, *Pediococcus*, *Pichia*, *Ogataea*, *Lactobacillus*, *Candida*, and *Wickerhamomyces* (in decreasing order of relative abundances) became prevalent, with *Brettanomyces* and *Pediococcus* being most prevalent at the end of the maturation phase (month 24). The relative abundances of the *Acetobacter* species varied during the maturation phase (between 1 and 50% of the reads).

##### Taxonomic analysis based on metagenomic recruitment plotting

**FIGURE 2:**
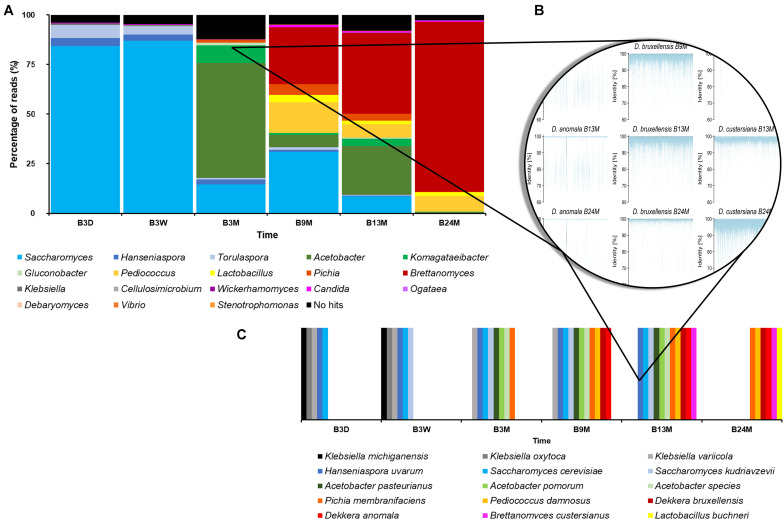
**(A)** Overview of the percentage of all reads as recruited by a certain genus of the six metagenomic sequence data sets, representing fermentation and maturation samples withdrawn after 3 days (B3D), 3 weeks (B3W), 3 months (B3M), 9 months (B9M), 13 months (B13M), and 24 months (B24M) of a lambic beer production process carried out in wooden casks. **(B)** Illustration of species-level taxonomic analysis by the construction of metagenomic recruitment plots, using genera that were represented by more than 0.1% of all metagenomic sequence reads. **(C)** Overview of the species-level taxonomic analysis data, as found by metagenomic recruitment plotting.

As the different taxonomy profiling tools gave slightly differing taxa and varying percentages of metagenomic sequence reads, metagenomic recruitment plotting was used to get a more consensus taxonomic classification. The metagenomic recruitment plots were constructed by aligning the metagenomic sequence reads to a custom-made database. This database consisted of genome sequences of all type strains belonging to those genera that were represented by more than 0.1% of all sequence reads, as found with any BLAST-based method, in any of the six metagenomes. These genera were *Acetobacter*, *Brettanomyces*, *Candida*, *Cellulosimicrobium*, *Debaryomyces*, *Gluconobacter*, *Hanseniaspora*, *Klebsiella*, *Komagataeibacter*, *Komagataella*, *Lactobacillus*, *Ogataea*, *Pediococcus*, *Pichia*, *Saccharomyces*, *Stenotrophomonas*, *Torulaspora*, *Vibrio*, and *Wickerhamomyces*. For the data sets of B3D, B3W, B3M, B9M, B13M, and B24M, 96.05, 95.30, 87.78, 95.04, 91.97, and 97.25% of the metagenomic sequence reads could be recruited by the concatenated genome sequences, respectively (Figure 2). These high percentages of total recruited reads seemed to underline that the reads attributed to *Brettanomyces/Dekkera* were underestimated in the profiling tools that use the databases nt, RefSeq, and nr.

The sequences of *A. pasteurianus*, *Acetobacter pomorum*, *Brettanomyces custersianus*, *Dekkera anomala*, *D. bruxellensis*, *Hanseniaspora uvarum*, *Klebsiella michiganensis*, *Klebsiella oxytoca*, *Klebsiella variicola*, *Lactobacillus buchneri*, *P. damnosus*, *Pi. membranifaciens*, *S. cerevisiae*, and *S. kudriavzevii* recruited metagenomic reads from at least one sample, throughout their lengths, mostly with 100% identity (Figure 2). The genome sequences of various *Acetobacter* species also recruited metagenomic reads of different samples throughout their lengths. However, the majority of these sequence identities varied between 80 and 90%, indicating the presence of at least one species in the samples that was not identical but very similar to various species belonging to the *Acetobacter* genus.

Through metagenomic recruitment plotting, it became clear that not only genera, as mentioned above, but also species within these different genera shifted during the lambic beer production process examined. At the beginning of the production process, the enterobacterial phase was characterized by the presence of *K. michiganensis*, *K. oxytoca*, and *K. variicola*, whereas the main fermentation phase was represented by *S. cerevisiae*. However, when the fermentation proceeded, more metagenomic sequence reads were attributed to *S. kudriavzevii*, showing at least a shared prevalence of both species from week 3 onward. The acidification phase was characterized by the presence of *A. pasteurianus*, *A. pomorum*, *P. damnosus*, and at least one more *Acetobacter* species. At the end of the maturation phase (B24M sample), the species representing the *Brettanomyces* genus shifted from *D. bruxellensis* to *B. custersianus*.

#### Functional Analysis of the Lambic Beer Metagenomes

**FIGURE 3:**
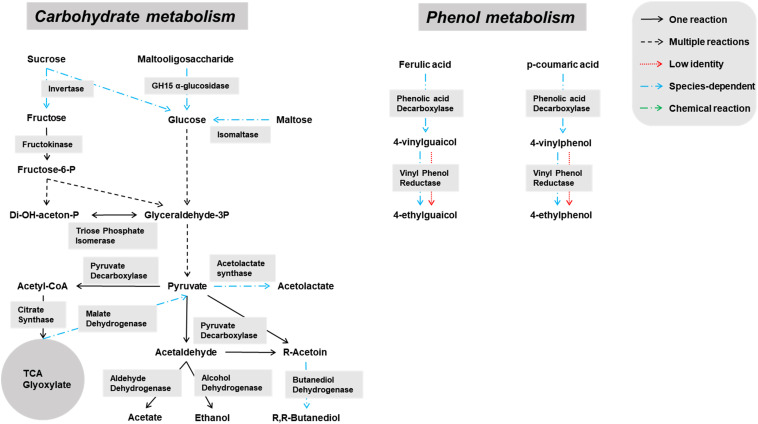
Overview of the main catabolic pathways reconstructed with the yeast-associated genes found through functional analysis of the six metagenomes of a 24-month lambic beer production process carried out in wooden casks. The yeast species included to construct the pathways were *Hanseniaspora uvarum, Saccharomyces cerevisiae*, *Saccharomyces kudriavzevii*, *Pichia membranifaciens*, *Dekkera bruxellensis*, and *Brettanomyces custersianus*. Catabolic reactions present in all these yeast species are indicated with black arrows. Catabolic reactions that were not present in all these yeast species are indicated with blue arrows. Non-enzymatic reactions are indicated with green arrows. Catabolic pathways represented by genes present at low identities are indicated with red arrows.

**FIGURE 4:**
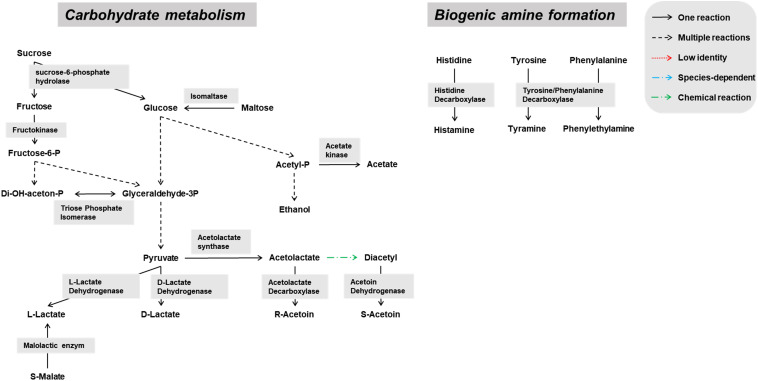
Overview of the main catabolic pathways reconstructed with the *Acetobacter*-associated genes found through functional analysis of the six metagenomes of a 24-month lambic beer production process carried out in wooden casks. Catabolic reactions present in all the *Acetobacter* species are indicated with black arrows. Non-enzymatic reactions are indicated with green arrows.

**FIGURE 5:**
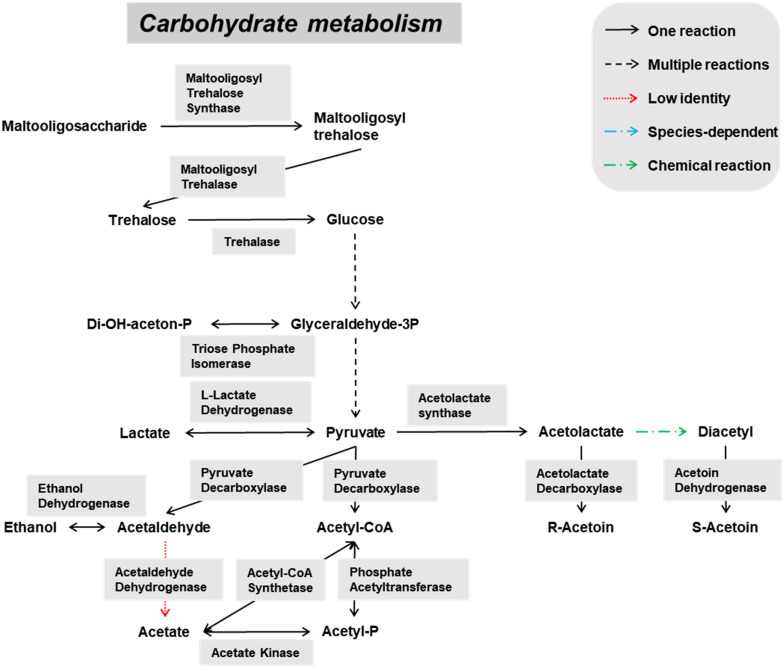
Overview of the main catabolic pathways constructed with the *Pediococcus damnosus*-associated genes found through functional analysis of the six metagenomes of a 24-month lambic beer production process carried out in wooden casks. Catabolic reactions present in *P. damnosus* are indicated with black arrows. Non-enzymatic reactions are indicated with green arrows.

Genes encoding enzymes of the general carbohydrate metabolism, in particular the Embden-Meyerhof-Parnas (EMP) pathway and the pentose phosphate (PP) pathway, were found on contigs assigned to all microbial groups abundantly present during the lambic beer production process examined, namely yeasts, AAB, and LAB (Figures 3–5). In particular, genes encoding enzymes necessary for the conversion of pyruvate into ethanol and acetic acid were associated with all yeast species found. Genes encoding the enzymes necessary for acetate formation from acetyl-P via pyruvate were attributed to different *Acetobacter* species. The entire homo- and heterolactic fermentation pathways, the genes for both D- and L-lactate dehydrogenases to convert pyruvate into lactic acid, and enzymes necessary for ethanol and acetate formation were attributed to *P. damnosus*. Further, genes encoding most of the enzymes involved in the tricarboxylic acid (TCA) cycle were attributed to the abundant yeast and *Acetobacter* species (Figures 3, 4). In this regard, genes encoding both a cytoplasmic malate dehydrogenase (involved in the TCA cycle) and a mitochondrial malate dehydrogenase (related with the glyoxylate pathway) were attributed to all yeast species, except for *H. uvarum* that only possessed the gene encoding a cytoplasmic one.

Some of the yeast enzymes involved in those metabolic pathways seemed to be species-dependent (Figure 3). More specifically, invertase-encoding genes and several genes coding for isomaltase were attributed to *S. cerevisiae*, *S. kudriavzevii*, *D. bruxellensis*, and *B. custersianus*, whereas genes encoding GH15 α-glucosidases that possibly break down maltooligosaccharides were attributed to *D. bruxellensis*, *B. custersianus*, and *S. kudriavzevii*, besides α-glucosidases degrading di- and trisaccharides. A gene encoding polygalacturonase was attributed to *S. cerevisiae* and *S. kudriavzevii*, indicating the possible pectinolytic potential of both yeast species. One specific gene encoding a β-glucosidase (NCBI accession number EIF48743) was attributed to *D. bruxellensis* (97% identity), indicating hydrolysis of specific β-glycosides, such as β-1,3 glucan laminarin. In contrast, another β-glucosidase that hydrolyses cellobiose, gentiobiose, and arbutin (NCBI accession number EIF45415) yielded no hits for *D. bruxellensis*. Both β-glucosidase genes were, at low identities, also present in *B. custersianus* (both 40% identity), and *H. uvarum* (51 and 61% identity, respectively), which indicated the presence of another β-glucosidase in both yeast species.

Due to a sucrose-6-phosphate hydrolase gene attributed to *P. damnosus*, this LAB species is also likely capable of splitting sucrose into glucose and fructose. Moreover, since a gene coding for a phosphotransferase system component was located in the vicinity of that sucrose-6-phosphate hydrolase gene, it is likely that *P. damnosus* imports sucrose into the cell as such to further hydrolyze its phosphorylated form. Next to many genes coding for GH13 family proteins (including α-1,4-glucosidases that use maltose and maltotriose as substrates) that were attributed to different yeast species, one GH13 gene was specifically attributed to *P. damnosus* and possibly encoded a glucan-hydrolyzing enzyme, whereas two maltose phosphorylase genes might enable the use of maltose.

The *Acetobacter* species present seemed to miss the complete EMP pathway, as the genes coding for glucose-6-phosphate isomerase and phosphofructokinase were absent. However, the genes for the entire PP pathway as well as a gene coding for pyruvate decarboxylase, which is important for the use of lactic acid as energy source, were attributed to these species. Furthermore, it turned out that *Acetobacter* species possessed genes coding for maltooligosyl trehalose synthase, enabling the degradation of maltooligosaccharides by their conversion into the non-reducing saccharide maltooligosyl trehalose (Figure 4). Furthermore, genes encoding trehalases (including periplasmic ones) were attributed to the *Acetobacter* species. Since the main characteristic feature of the genus *Acetobacter* is the oxidation of ethanol by pyrroloquinoline quinone-dependent membrane-bound dehydrogenases, the genes encoding the three subunits of the alcohol dehydrogenase and aldehyde dehydrogenase, as well as genes encoding NADH-quinone oxidoreductase, were attributed to the *Acetobacter* species present. Cytoplasmic NADH-dependent dehydrogenases were also encoded in their genomes. Finally, genes encoding specific esterases, namely EST1 (Swiss-Prot accession number O66374) and EST2 (Swiss-Prot accession number O66382), were attributed to some *Acetobacter* species, including *A. pasteurianus* (both 89% identity).

Species specificity was also encountered in genes coding for phenolic acid decarboxylase, which were attributed to *D. bruxellensis* (98% identity to UniProt accession number A0A1L5YR15), *S. cerevisiae* (99% identity to Swiss-Prot accession number Q03034), and *P. damnosus* (83% identity to UniProt accession number P94900). Genes coding for superoxide dismutase, which can also display vinyl phenol reductase activity, were attributed to *B. custersianus* (89% identity to UniProt accession number I2JWC1), *D. bruxellensis* (100% identity to UniProt accession number I2JWC1), *S. cerevisiae* (73% identity to UniProt accession number I2JWC1), *S. kudriavzevii* (75% identity to UniProt accession number I2JWC1), and *H. uvarum* (64% identity to UniProt accession number I2JWC1).

*Pediococcus damnosus* present in the lambic beer metagenomes could convert L-malic acid into L-lactic acid via the malolactic enzyme, the gene of which was attributed to this LAB species (Figure 5). Genes encoding malic enzyme, which could convert malic acid through pyruvate into lactic acid, were attributed to all yeast species. Concerning acetoin production, genes encoding α-acetolactate synthase and α-acetolactate decarboxylase, acting on pyruvate, and diacetyl reductases, acting on α-acetolactate, could be attributed to both *P. damnosus* and *Acetobacter* species. Genes encoding pyruvate decarboxylase, converting pyruvate into acetoin, could be attributed to all yeast species present too.

Also several genes coding for the hop resistance proteins HitA (100% identical to Swiss-Prot accession number Q93V04), HorA (100% identical to UniProt accession number G8PFG1), HorB (100% identical to UniProt accession number A4UX85), and HorC (94% identical to UniProt accession number A4UX86), as well as a gene encoding a putative glucan synthase (97% identical to NCBI accession number ABB51206) supposedly responsible for exopolysaccharide formation and a ropy phenotype, were attributed to *P. damnosus*. However, slime formation did not occur during the fermentation or maturation phases of the lambic beer production process examined.

Biosynthetic pathways for biogenic amine formation were found as well. Part of a gene coding for a lysine decarboxylase (Swiss-Prot accession number P52095.2), and genes coding for a histidine decarboxylase (Swiss-Prot accession number P00862) and phenylalanine/tyrosine decarboxylase (UniProt accession number Q1JTV5, NCBI accession number AAN77279.2) were attributed to the enterobacterial genus *Klebsiella* and the LAB species *P. damnosus*, respectively. These decarboxylases are responsible for cadaverine, histamine, and tyramine formation, respectively. Genes coding for arginine decarboxylase (Swiss-Prot accession number P21885) and aromatic-L-amino-acid decarboxylase (UniProt accession number I0DFJ0) were not found.

### Metabolite Target Analyses

**FIGURE 6:**
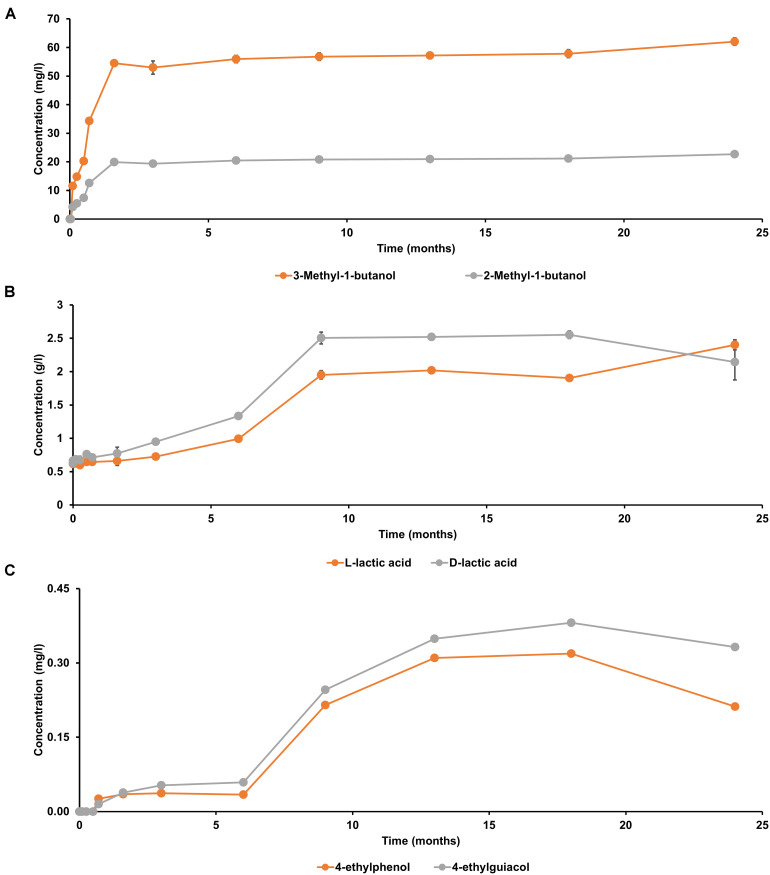
Production of **(A)** 2-methylbutanol and 3-methylbutanol, **(B)** D-lactic acid and L-lactic acid, and **(C)** 4-ethylguaiacol and 4-ethylphenol during a 24-month lambic beer production process carried out in wooden casks. The error bars represent standard deviations of technical replicates.

Both D-lactic acid and L-lactic acid were present in a nearly equimolar ratio in the initial wort (Figure 6), reflecting the manual acidification of the wort with lactic acid at the start of the lambic beer production process followed. In contrast, 2-methylbutanol, 3-methylbutanol, 4-ethylguaiacol, and 4-ethylphenol were not present from the start of the fermentation process. The yeast-associated metabolites 2-methylbutanol and 3-methylbutanol were produced in a constant ratio during the main fermentation phase (first 7 weeks). From month 3 of the lambic beer production process onward, D-lactic acid and L-lactic acid were produced in nearly equal concentrations, indicating the start of the acidification phase, which was characterized by the prevalence of LAB. The concentration decrease of mostly D-lactic acid could indicate stereospecific consumption of lactic acid during the later stages of the lambic beer production process. From 6 months onward, until the end of the maturation phase, the phenolic compounds 4-ethylguaiacol and 4-ethylphenol were produced, in accordance with the presence of *Brettanomyces* yeasts during the maturation phase. 4-Ethylguaiacol was produced in higher concentrations than 4-ethylphenol, as shown through mass spectrometric analysis. For all other metabolite concentrations, see De Roos et al. (2018a, b).

### Alpha-Diversity and Correlation Analysis Between Microbial Species and Metabolites

**FIGURE 7:**
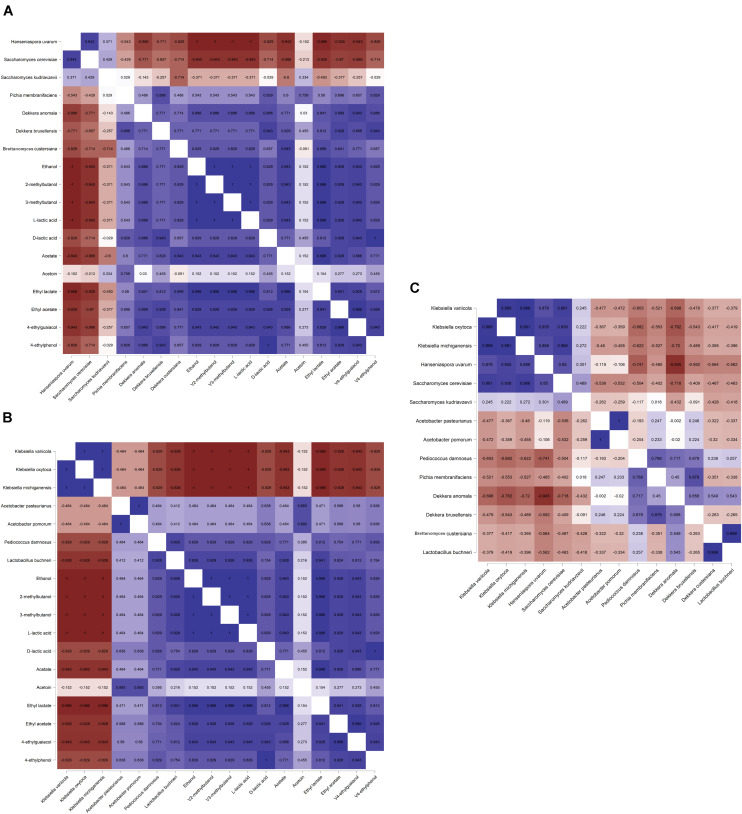
Spearman correlation analysis (*P* < 0.05) between the different yeast **(A)** and bacterial **(B)** species (based on metagenomic recruitment plotting) and the concentrations of dedicated metabolites produced as well as between **(C)** the different microbial species during a 24-month lambic beer production process carried out in wooden casks. Positive correlations are depicted in blue, negative correlations are depicted in red. Data for ethanol, acetate, acetoin, ethyl acetate, and ethyl lactate concentrations were taken from De Roos et al. (2018a, b). Color intensities represent the value of the correlation coefficients with |1| depicting the strongest intensity.

**FIGURE 8:**
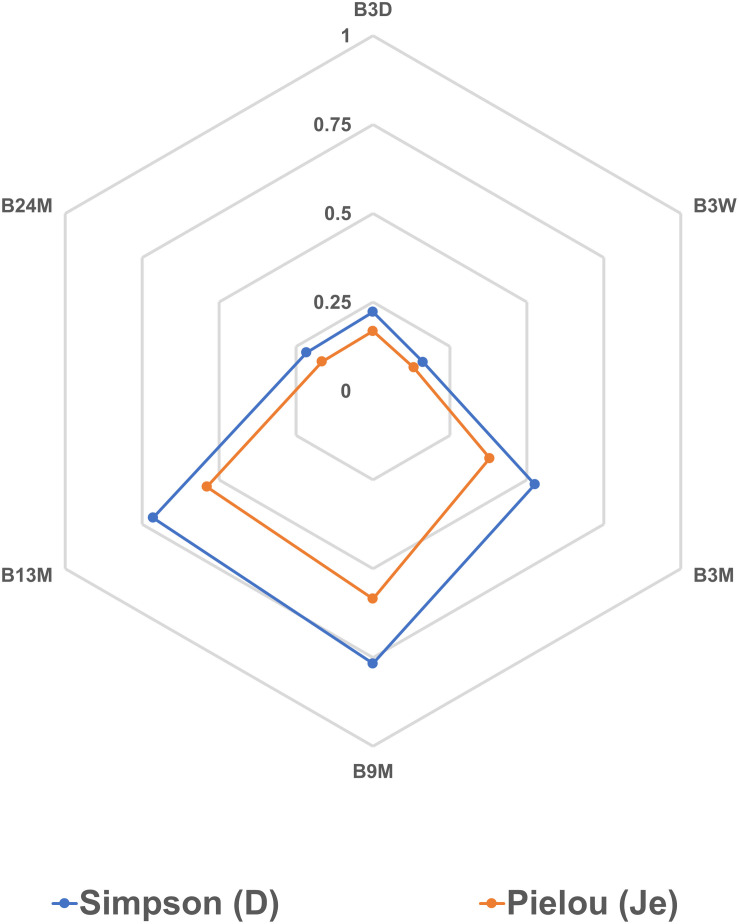
Alpha-diversity metrics based on the relative abundances of bacterial and yeast genera obtained by metagenomic recruitment plotting applied to samples from the 24-month lambic beer production process carried out in wooden casks. The Simpson (D) and Pielou (Je) indexes were calculated for all samples to measure their diversity and evenness, respectively. The sample codes represent fermentation and maturation samples withdrawn after 3 days (B3D), 3 weeks (B3W), 3 months (B3M), 9 months (B9M), 13 months (B13M), and 24 months (B24M).

The Spearman correlation analysis showed significant correlations between different microbial species (Figures 7A–C), reflecting the different microbial phases during the lambic beer production process of the present study. However, it failed to show significant, biologically relevant correlations between the different species present and the different metabolites produced. Yet, *S. cerevisiae*, *H. uvarum*, *K. michiganensis*, *K. variicola*, and *K. oxytoca*, which were all mostly present during the beginning of the fermentation process, were significantly positively correlated and reflected the enterobacterial and main fermentation phases. *Acetobacter pasteurianus* and *A. pomorum*, which were mostly present at month 3 of the lambic beer production process, were also significantly positively correlated and reflected the acidification phase. Furthermore, *P. damnosus* and *L. buchneri*, which appeared later during the acidification phase, were significantly positively correlated too. However, *L. buchneri* was only present in low relative abundance and mostly appeared at the end of the lambic beer production process examined. Finally, *D. bruxellensis* and *P. membranifaciens* were the main yeast species present during the maturation phase and were significantly positively correlated. As shown by the alpha-diversity metrics (Figure 8), the microbial diversity and evenness was low during the main fermentation phase (samples B3D and B3W) and increased during the acidification phase (B3M and B9M). This was reflected by the high prevalence of the *Saccharomyces* genus during the first weeks of fermentation and the more even presence of other, different microbial genera (*Saccharomyces*, *Acetobacter*, *Komagataeibacter*, *Pichia*, *Pediococcus*, and *Brettanomyces*) during the acidification phase. During the maturation phase (B13M and B24M), when the environmental conditions became harsher, the microbial diversity and evenness decreased again, evolving toward the high-prevalence of one genus namely *Brettanomyces* (Figure 2).

## Discussion

Former studies on lambic beer production processes focused mostly on the different microorganisms and metabolites present during the four consecutive phases of the fermentation and maturation process (Van Oevelen et al., 1977; Verachtert and Dawoud, 1984; Verachtert et al., 1989; Martens et al., 1991; Shanta Kumara and Verachtert, 1991; De Roos et al., 2018a, b). In particular, the most recent studies have hypothesized possible reasons behind particular community dynamics of AAB, LAB, and yeasts during the main fermentation, acidification, and maturation phases of lambic beer production processes. Moreover, some new fermentation characteristics have been shown, such as the occurrence of a malolactic fermentation most likely carried out by *P. damnosus*, acetoin production by *Acetobacter* species, the possible consumption of acetoin by *Brettanomyces* species, and the simultaneous breakdown of maltooligosaccharides probably by yeast species present at low counts (De Roos et al., 2018a). However, to strengthen these results and to clarify some of the hypotheses proposed in said studies, a temporal metagenomic analysis of a lambic beer production process that has been investigated microbiologically and metabolically in detail before was performed to target both the taxonomy and the functional properties of the different microorganisms involved.

The taxonomic analysis of the six metagenomes of the 24-month lambic beer production process of the present study confirmed the succession of different microbial groups during four distinct phases, with a rather restricted bacterial and yeast species diversity especially during the main fermentation and maturation phases, as shown by the alpha-diversity metrics. These four consecutive phases encompassed a short enterobacterial phase, spanning the first week of fermentation only, a main alcoholic fermentation phase represented by *Saccharomyces* species, an acidification phase represented by AAB species (*A. pasteurianus*, *A. pomorum*, and an unknown *Acetobacter* species) and LAB species (*P. damnosus*), and a maturation phase represented by LAB species (*P. damnosus* and *L. buchneri*) and *Brettanomyces* species. This was indeed in accordance with the temporal culture-dependent data obtained for the lambic beer production process of the present study that have been published before (De Roos et al., 2018a, b).

Concerning the AAB, the genomes of some *Acetobacter* species rarely recruited reads with identities over 90% through metagenomic recruitment plotting. Hence, since all the available genomes belonging to *Acetobacter* species were included in this taxonomic analysis, an *Acetobacter* species whose genome sequence was not available or a new *Acetobacter* species had to be present in the lambic beer ecosystem examined. However, based on the culture-dependent data of the lambic beer production process of the present study, the metagenomic sequence reads most likely belonged to the species *A. lambici* (De Roos et al., 2018b). *Acetobacter lambici* was first isolated from a lambic beer production process and has not been isolated from other ecosystems up to now, indicating a possible niche specificity (Spitaels et al., 2014a). Species belonging to the *Acetobacter* genus were most likely responsible for the substantial production of acetoin during lambic beer maturation, due to their oxidation of lactic acid, as has been shown phenotypically before in both lambic beer production and cocoa fermentation processes (Adler et al., 2014; Moens et al., 2014; De Roos et al., 2018b). The presence of acetolactate synthase and acetolactate decarboxylase genes belonging to the AAB species identified supported this biosynthesis potential. Although many of the microbial species found, encompassing *P. damnosus* and various yeast species, possessed the necessary genes for acetoin production, the fact that the production coincided with the appearance of AAB and that more acetoin was produced at the air/liquid interface of the casks underlined the production of acetoin by the AAB (De Roos et al., 2018b). Besides in the production of acetoin, the AAB could also play a role in ethyl acetate formation, due to the presence of an EST1 esterase, the activity of which has been shown before (Kashima et al., 2000).

With regard to the LAB, the presence of *P. damnosus* in high relative abundances could be hold responsible for a fast consumption of malic acid during the acidification phase, as this has been shown phenotypically before (De Roos et al., 2018a). However, culture-dependent analysis is limited in addressing sub-dominant microbial groups, therefore making it difficult to address malic acid consumption to *P. damnosus* solely. Yet, the metagenomic analysis of the present study disclosed *P. damnosus* as the only LAB species present at the moment of malic acid consumption. This was further strengthened by the measurement of a nearly equimolar production of D-lactic acid and L-lactic acid and the attribution of both D-lactate and L-lactate dehydrogenase genes to *P. damnosus*. Moreover, it was the only species found in the metagenome to possess the malolactic enzyme. Although yeast species are also capable of converting malic acid into lactic acid via a malic enzyme, it was highly unlikely that yeast species were responsible for the malic acid consumption here, as their appearance in the lambic beer production process did not coincide with the phase of malic acid consumption. Although only L-lactic acid can be formed from L-malic acid by the malolactic enzyme, this was not reflected by the metabolite measurements of D-lactic acid and L-lactic acid. However, due to the low concentration of malic acid present in the wort, differences in L-lactic acid concentrations coming from the conversion of L-malic acid are difficult to spot. Pure culture fermentations could be performed to further confirm malolactic fermentation by *P. damnosus*. Further, *P. damnosus* was most probably responsible for biogenic amine production during the lambic beer production process examined, as this LAB species possessed the necessary decarboxylase genes for the production of histamine and tyramine. Moreover, biogenic amines are produced during the acidification phase of a lambic beer production process, as has been shown before for the process examined (De Roos et al., 2018a). Further, the partial lysine decarboxylase gene belonging to *Klebsiella* species was in accordance with the low concentrations of cadaverine produced during the first week of the lambic beer production process of the present study (De Roos et al., 2018a). However, the concentrations of these biogenic amines are very low (De Roos et al., 2018a). Besides biogenic amine production, some *Klebsiella* species, such as *K. oxytoca* can be considered as emerging pathogens (Singh et al., 2016). However, *Klebsiella* species were only present in low numbers during the first week of the lambic beer production process and were not detected during later fermentation and maturation stages.

Concerning the yeasts, the prevalence of *Brettanomyces* species during the maturation phase was responsible for the typical Brett flavor of lambic beer, which is mostly determined by the volatile phenolic compounds 4-ethylguaiacol and 4-ethylphenol (Lentz and Harris, 2015; Steensels et al., 2015). Both phenolic compounds are produced by the consecutive activity of the enzymes phenylacrylic/phenolic acid decarboxylase and a vinyl phenol reductase/superoxide dismutase. The absence of phenolic acid decarboxylase in *B. custersianus* could explain the lack of conversion of ferulic acid and *p*-coumaric acid into their respective vinyl or ethyl derivatives (Harris et al., 2008). Moreover, as phenolic acid decarboxylase is used for the detoxification of hydroxycinnamic acid, it could be one of the reasons for the prevalence of *D. bruxellensis* at the beginning of the maturation phase of the lambic beer production process examined. A vinyl phenol reductase/superoxide dismutase was found in the different yeast species identified. However, vinyl phenol reductase activity is generally only encountered in species that belong to the *Brettanomyces* genus (Granato et al., 2015). The presence of certain cofactor-binding structural features in amino acid sequences of superoxide dismutases may explain the presence of vinyl phenol reductase activity in these yeasts, The production of 4-ethylguaiacol and 4-ethylphenol that coincided with the presence of the *Brettanomyces* species seemed to underline this limited occurrence of vinyl phenol reductase activity. The exact role of this activity in yeasts remains unclear. However, when cells are introduced in an anaerobic environment, it can aid in maintaining the redox balance by using NADH + H^+^ as cofactor to reduce hydroxystyrenes to their corresponding ethyl derivatives (Steensels et al., 2015). 4-Ethylguaiacol and 4-ethylphenol are mainly produced under conditions with little residual carbohydrates (Chatonnet et al., 1995; Lentz and Harris, 2015). In addition, genetic diversity studies have revealed substantial genotypic inter-strain variability within *D. bruxellensis*, showing, for example, that brewing strains display a more efficient metabolism of ferulic acid over *p*-coumaric acid (Lentz and Harris, 2015). This specificity of brewing strains could be visible in the higher concentrations of 4-ethylguaiacol as compared to 4-ethylphenol found in the maturing lambic beer of the present study. Nevertheless, the higher production of 4-ethylguaiacol as compared to 4-ethylphenol could also be due to differences in concentrations of the precursor compounds present in the different matrices rather than interspecific variations and adaptation of the *Brettanomyces* species. In wine, the reverse ratio is often found, which may be a reason why *Brettanomyces* is perceived as an off-flavor-producing yeast during wine fermentation; in contrast, *Brettanomyces* is desirable for its Brett flavor contribution during lambic beer production (Romano et al., 2008; Vanbeneden et al., 2008). Moreover, this correlation between genotypic groups of *D. bruxellensis* and their source of isolation was also true for the absence of a dedicated β-glucosidase. This finding seems to represent a common trait shared by beer strains, as they are the only strains that are not capable of degrading specific β-bonds, such as β-1,4 bonds (cellobiose) and β-1,6 bonds (gentiobiose), due to the absence of the gene for this specific β-glycosidase, compared with wine and soft drink strains of *D. bruxellensis* (Crauwels et al., 2014, 2015). However, these beer strains possess another dedicated β-glucosidase gene that is capable of hydrolyzing β-1,3 glycosidic bonds, which are found in the β-glucans present in yeast cell walls and cereals. The *D. bruxellensis* species occurring during the lambic beer production process of the present study did contain this β-glucosidase gene. This may directly impact the flavor production ability of strains belonging to this species by elaborating β-1,3 glycosidic-bound aroma compounds as well as improving their competitive fitness toward other microorganisms. Also, some *P. damnosus* species can synthesize a β-D-glucan with β-1,3 glycosidic bonds (Llaubères et al., 1990). Especially during the warm summer months, LAB can produce exopolysaccharides in the fermenting wort or maturing beer (Van Oevelen et al., 1977; Van Oevelen and Verachtert, 1979). The presence of this β-1,3 glucosidase could explain previous observations that when LAB and *Brettanomyces* species occurred together during the first months of the maturation phase a more pronounced over-attenuation occurred (Shanta Kumara and Verachtert, 1991). Moreover, together with the species diversity of different lambic beer productions, the presence of this dedicated β-glucosidase gene could explain why slime formation occurs in some lambic beer production processes and not in others and why it usually disappears later on in the production process.

Although previously ascribed to *Brettanomyces* species, the breakdown of maltooligosaccharides during the lambic beer production process investigated started already early in the fermentation phase, namely before the appearance of these yeasts (Shanta Kumara et al., 1993; De Roos et al., 2018a). Moreover, maltooligosaccharide degradation happened continuously and simultaneously for all chain lengths up to a polymerization degree of eight, as shown phenotypically before (De Roos et al., 2018a). Therefore, the breakdown of maltooligosaccharides during the early stages of the fermentation process was most likely due to the presence of yeast species at low counts or as a result of cell lysis of yeasts present during the main fermentation phase, thereby releasing dextrin-degrading enzymes into the fermenting wort. Indeed, *S. kudriavzevii* possessing an α-glucosidase capable of maltooligosaccharide degradation appeared at the moment that maltooligosaccharide degradation was initiated. In this regard, the AAB species examined possessed genes encoding maltooligosyl trehalose synthase and maltooligosyl trehalase, allowing them to metabolize maltooligosaccharides through their conversion into trehalose, which could be further metabolized via the general AAB carbohydrate metabolism. This pathway could be a way for AAB to use the dextrins available during the later stages of the lambic beer production process, when mono- and disaccharides are depleted. Further, it could be a mechanism to obtain their necessary intracellular carbon, besides through the consumption of lactic acid, of which acetoin is an overflow metabolite (Adler et al., 2014; Moens et al., 2014). Finally, it may partly explain the growth of *A. pasteurianus* during the acidification phase under appropriate environmental conditions (De Roos et al., 2018b). However, whether or not AAB do so needs to be further investigated.

## Conclusion

In conclusion, the temporal analysis of six metagenomes from a 24-month lambic beer production process carried out in a Belgian traditional lambic brewery confirmed the occurrence of a microbial succession during four distinct phases. Moreover, only well-adapted microbial species could thrive throughout the fermentation and maturation process, as shown culture-dependently before, due to the harshness and specific composition of the fermenting and maturing lambic beer matrix. This limited microbial diversity was reflected by the few species retrieved from the different metagenomic samples. Due to the small sample size, multiple comparisons between microorganisms and metabolites rendered poorer results than expected, which is, however, a well-known challenge in the bioinformatic field (Noecker et al., 2019). Although the limited sample size hampered a rigorous statistical analysis, the functional analysis of the metagenomic data using a single-microbial species approach provided an added value and could link the consumption of substrates and the production of dedicated metabolites during the lambic beer production process examined to specific microorganisms. Some of these functional properties, such as acetoin formation, β-glucosidase activity, and vinyl phenol reductase activity could have economic repercussions, as they directly influence the aroma and taste profile of the end-products. In this regard, some of the *Brettanomyces* species present were found to lack the necessary enzymatic equipment to produce 4-vinylguaiacol and 4-vinylphenol, which are the precursor compounds responsible for the typical Brett flavor of lambic beers. This better understanding of the microbial dynamics behind these fermentation characteristics could improve brewery practices to assure more quality-stable end-products. Future metagenomic studies will be performed, encompassing a higher number of samples as well as replicates, enabling the functional characterization of the microbial communities throughout the entire lambic beer production process in more detail. This will also allow to do more rigorous statistical analysis and further investigate the different specific fermentation characteristics and pathways introduced in this paper.

## Data Availability Statement

The datasets generated for this study can be found in the online repositories. The names of the repository/repositories and accession number(s) can be found at: https://www.ebi.ac.uk/metagenomics/, PRJEB28363.

## Author Contributions

JD contributed to the experimental work, the acquisition, processing, interpretation of the data, and the drafting of the manuscript. MV contributed to the experimental work, the acquisition, processing, and interpretation of the data. SW and LD contributed to the interpretation of the data, supervision of the work, and review and editing of the manuscript. All authors contributed to the article and approved the submitted version.

## Conflict of Interest

The authors declare that the research was conducted in the absence of any commercial or financial relationships that could be construed as a potential conflict of interest.

## Footnotes

1 https://www.ncbi.nlm.nih.gov/

2 http://hmmer.org/

3 www.cazypedia.org

4 http://www.ebi.ac.uk/ena/data/view/PRJEB28363
